# Fluorescent component and complexation mechanism of extracellular polymeric substances during dye wastewater biotreatment by anaerobic granular sludge

**DOI:** 10.1098/rsos.171445

**Published:** 2018-02-28

**Authors:** Na Li, Dong Wei, Qunqun Sun, Xiao Han, Bin Du, Qin Wei

**Affiliations:** 1 School of Resources and Environment, University of Jinan, Jinan 250022, People's Republic of China; 2 Key Laboratory of Interfacial Reaction and Sensing Analysis in Universities of Shandong, School of Chemistry and Chemical Engineering, University of Jinan, Jinan 250022, People's Republic of China

**Keywords:** biotreatment, methylene blue, anaerobic granular sludge, extracellular polymeric substances, excitation–emission matrix

## Abstract

In this study, methylene blue (MB) wastewater was biotreated by anaerobic granular sludge (AnGS), and the fluorescent components of extracellular polymeric substances (EPS) and complexation mechanism were evaluated. Based on the experimental data, the sorption of MB by both live and inactivated AnGS followed the pseudo-second-order model, and the adsorption isotherm conformed well to the Langmuir model. It was shown that the difference in the sorption of live and inactivated AnGS was not significant, indicating that the sorption is mainly a physical–chemical process and metabolically mediated diffusion is negligible. The interaction between EPS and MB was proved by three-dimensional excitation–emission matrix (3D-EEM) and synchronous fluorescence spectra. 3D-EEM indicated that protein (PN)-like substances were the main peaks of EPS, and gradually quenched with increase of MB concentrations. According to synchronous fluorescence spectra, the main fluorescence quenching was caused by PN-like and humic-like fractions, and belonged to the static type of quenching. FTIR spectra demonstrated that hydroxyl and amino groups played a major role in MB sorption.

## 1. Introduction

Dyes are widely used in industries such as textiles, leather, paper and plastics etc., in which they are regarded as the predominant contaminants in water sources [1]. The pollution of dyes in the environment is a matter of concern owing to their toxicity to many life forms. The discharge of dyes in the environment is worrying for both toxicological and aesthetic reasons [2,3]. As one kind of basic dye, methylene blue (MB) is commonly applied for dying cotton, wool and silk. Exposure to MB can cause eye burns, and if inhaled, it may cause nausea, vomiting, diarrhoea, gastritis and breathlessness [4,5]. To date, the treatment of dye wastewater is commonly carried out using biological and physico-chemical technologies, including biosorption, membrane filtration, ozonation, photocatalysis, advanced oxidation, electrochemical treatment and degradation [6–8].

Biological treatment expresses the most attractive features for the elimination of dye effluent since it is relatively low-cost and environmentally friendly, especially under anaerobic conditions [9]. It is well accepted that anaerobic granular sludge (AnGS) provides a number of significant advantages because of its unique granular attributes, such as excellent settle ability, higher biomass concentration and good treatment performance. Ong *et al*. [9] evaluated the biodegradation of MB wastewater by using an up-flow anaerobic sludge blanket reactor (UASB), finding that more than 90% of colour removal efficiency was achieved in the UASB reactor with 0.627 mmol l^−1^ of MB concentration. Moreover, AnGS is also regarded as an effective biosorbent in the treatment of dye contaminated environments owing to its abundant sorption sites. Liu *et al*. [10] investigated the effect of functional groups on the adsorption process of MB on AnGS, suggesting that carboxyl and amino groups were identified as the most important moieties involved in the binding process. Wang *et al*. [11] observed that anaerobic sludge had a much higher equilibrium adsorption density on Rhodamine B than on Eosin Y. Therefore, it is essential to study both biodegradation and biosorption processes of MB in the AnGS system to fully evaluate the fate of MB.

Additionally, extracellular polymeric substances (EPS) are well reported to play a crucial role in the formation and stability of microbial structure [12]. They are regarded as complex high-molecular-weight extracellular polymers, including polysaccharides, proteins and nucleic acids etc. [13]. Since many functional groups are present in the EPS matrix, such as carboxyl, phosphoric, amine and hydroxyl groups, EPS have been proved to contribute to binding of dye contaminants in the adsorption process [14]. Hence, information on EPS components and their complexation mechanism with dye during its biotreatment process should be investigated in depth. To date, the fluorescence approach has been successfully applied for the characterization of the binding mechanism between EPS and dye, such as using three-dimensional excitation–emission matrix (3D-EEM) and synchronous fluorescence. 3D-EEM is regarded as a rapid, selective and sensitive monitoring tool to analyse EPS fluorescence components [15], whereas synchronous fluorescence spectroscopy could supply information on the multi-components present in EPS without pre-separation. Therefore, the application of fluorescence analysis is expected to be a useful method for the understanding of dye wastewater biotreatment by AnGS.

In the present study, the biodegradation and biosorption of MB removal were evaluated through study of the adsorption isotherm for live and inactivated AnGS. The fluorescent components of EPS and complexation mechanism during MB wastewater biotreatment by AnGS were also investigated. The interaction between EPS and MB was explained by using 3D-EEM fluorescence spectroscopy and synchronous fluorescence spectra. In addition, FTIR analysis was employed to investigate functional groups (e.g. carboxyl, amino), which were the main binding sites in the biosorption process.

## 2. Material and methods

### 2.1. Reagents and materials

Methylene blue (MB) used in this study was purchased from Sinopharm Chemical Reagent Beijing Co., Ltd., China. AnGS was obtained from a wastewater treatment plant treating high-strength industrial wastewater in Dezhou, Shandong province (treatment capacity 5000 m^3^ d^−1^). Figure S1 in the electronic supplementary material shows the appearance of the AnGS. The granules were globular and smooth-faced; the colour was dark brown and the average size was about 2 mm. Before the sorption experiment, AnGS was washed three times using deionized water to remove the surface soluble ions.

### 2.2. Biotreatment experiments

Inactivated AnGS was used to eliminate degradation, allowing only sorption to be studied. The inactivation procedure was modified according to the method used by Wang & Grady [16]. AnGS was inactivated using an autoclave at 120°C for 30 min. The sludge was then centrifuged and washed three times using deionized water and stored in a refrigerator at 4°C for further use.

The MB sorption experiments were carried out using approximately 0.15 g (dry weight) of live and inactivated AnGS at 20 ± 1°C, and the working volume of each Erlenmeyer flask was 50 ml. The initial pH of the mixed solution was adjusted to 7.0 by using 0.1 mol l^−1^ HCl and NaOH. The sorption isotherm experiment was done at different initial concentrations of MB ranging between 25 and 1800 mg l^−1^ and all batch biosorption tests were performed with 10 h of contact time to ensure equilibrium. For kinetic sorption tests, 0.15 g adsorbent was added to the solution of 1000 mg l^−1^ MB at a predetermined time (0–120 min). Each experiment was duplicated under identical conditions.

The amount of MB adsorbed onto the samples was calculated according to the following equation:

2.1 $$q_{t}=\frac{(c_{0}-c_{t})\times V}{m},$$

where *c*_0_ (mg l^−1^) and *c_t_* (mg l^−1^) are the initial concentration of adsorbate and the concentration at time *t* (min), respectively. *V* (l) stands for the volume of adsorbate solution, *m* (g) is the mass of adsorbents and *q_t_* (mg g^−1^) is the adsorbed amount at time *t* (min).

### 2.3. EPS extraction

EPS was extracted by using the heating method developed by Shen *et al*. [17]. The extraction process was as follows: AnGS was first washed three times with deionized water. Then, live AnGS was suspended in 50 ml deionized water and heated at 80°C in a water bath for 1 h. Finally, the solution was centrifuged at 10 000 rpm for 15 min. The supernatant, after filtering through a 0.45 µm pore size filter, was regarded as EPS of live AnGS.

### 2.4. Analytical methods

The fluorescence spectra of the EPS solution were measured with a luminescence spectrometer (LS-55, Perkin-Elmer Co., USA). EEM spectra were obtained by scanning over excitation wavelength (Ex) between 220 and 400 nm at 10 nm increments and emission wavelength (Em) between 250 and 580 nm at 10 nm increments, with a slit-width of 20 nm. Synchronous fluorescence spectra were recorded for samples prepared at different concentrations of MB with a constant offset (Δ*λ *= 60 nm) between excitation and emission wavelengths. The excitation and emission slits were set to 20 and 20 nm, respectively. Scan speed was 1200 nm min^−1^.

MB concentration was analysed using a UV-spectrophotometer (TU-1901, Beijing Purkinje General instrument Co., Ltd., China) at 665 nm. FTIR spectra were measured by using a PerkinElmer spectrometer (Spectrum One, USA) in the range of 4000–400 cm^−1^.

## 3. Results

### 3.1. Isotherm studies

**Figure 1.**
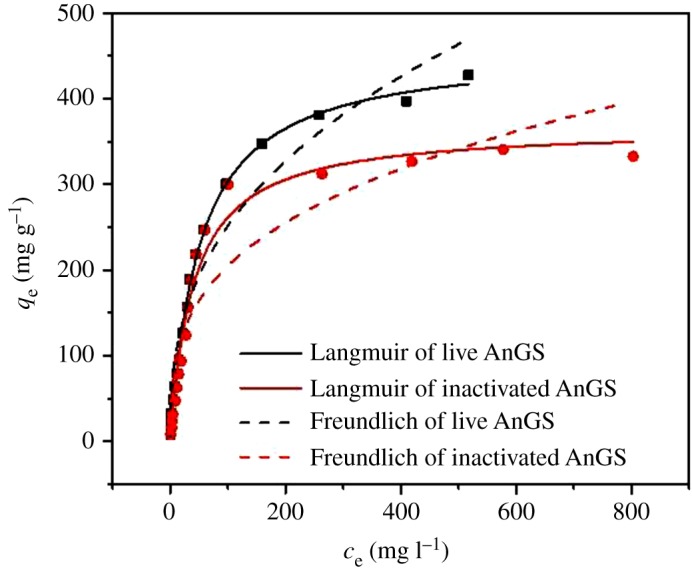
Sorption isotherms fit of MB with live and inactivated AnGS.

The adsorption isotherm models are characterized by certain constants, the values of which represent the surface properties and affinity of the biosorbent. Langmuir and Freundlich models were used to describe the MB adsorption onto live and inactivated AnGS. The results are shown in figure 1. These adsorption isotherms can be expressed as:

3.1 $$q_{e}=\frac{bq_{m}c_{e}}{1+bc_{e}}$$

and

3.2 $$q_{e}=K_{f}{c_{e}}^{1/n},$$

where *q*_e_ (mg g^−1^) and *c*_e_ (mg l^−1^) represent the amount of adsorbed MB and MB concentration at equilibrium, respectively; *q*_m_ (mg g^−1^) indicates the monolayer adsorption capacity of adsorbent and the Langmuir constant *b* (l mg^−1^) is related to the energy of adsorption; *K*_f_ (mg g^−1^) is the Freundlich constant related to adsorption capacity of adsorbent and 1/*n* is the Freundlich exponent related to adsorption intensity.

The obtained parameters from fitting Freundlich and Langmuir sorption isotherms are presented in table 1. The calculated maximum adsorption capacity (*q*_m_) of MB was found to be 458.33 mg g^−1^ for live AnGS and 367.47 mg g^−1^ for inactivated AnGS. It is also noted that the values of 1/*n* are 0.38 and 0.317 for live and inactivated AnGS, respectively. The values of 1/*n* are between 0.1 and 1 in the biosorption process, representing a favourable sorption, according to the paper reported by Ncibi *et al*. [18].

**Table 1.** The adsorption isotherm constants and correlation coefficients of MB adsorption onto live and inactivated AnGS.

	Langmuir equation			Freundlich equation		
adsorbent	*q*_m_ (mg g^−1^)	*b* (l mg^−1^)	*R*^2^	*K*_f_ (l g^−1^)	1/*n*	*R*^2^
live AnGS	458.33	0.020	0.99	43.73	0.38	0.94
inactivated AnGS	367.47	0.025	0.98	47.58	0.317	0.84

As shown in figure 1, MB sorption by both live and inactivated AnGS was better fitted to the Langmuir equation, which suggests the sorption took place at specific homogeneous reactive sites and was probably a monolayer process [19]. The data revealed that although the sorption constants obtained in this study for live and inactivated AnGS were very close, the sorptive capacity of the live AnGS was higher than that of the inactivated AnGS. MB sorption by live and inactivated AnGS includes two different processes. The first sorption process is the binding of MB to the cell surface, and is considered as biosorption. The second sorption process is the sorption of MB into the cell across the cell membrane, which is referred to as intracellular sorption or active sorption [20]. The first process takes place for both live and inactivated AnGS and the second process occurs only in live AnGS. Therefore, the higher sorptive capacity of the live AnGS could be attributed to the active sorption of the AnGS.

### 3.2. Kinetic studies

Removal kinetics of the adsorption process were studied with an initial MB concentration of 1000 mg l^−1^. In order to analyse the mechanism of the adsorption process, two different kinetic models were used for correlation of biosorption data.

The pseudo-first-order and pseudo-second-order kinetic models have the following forms:

3.3 $$\text{lg}(q_{e}-q_{t})=\mathrm{lg}q_{e}-\frac{k_{1}t}{2.303}$$

and

3.4 $$\frac{t}{q_{t}}=\frac{1}{k_{2}q_{e}^{2}}+\frac{t}{q_{e}},$$

where *q*_e_ and *q_t_* (mg g^−1^) are the amount of MB adsorbed at equilibrium and time *t*, respectively; *k*_1_ (min^−1^) and *k*_2_ (g (mg min)^−1^) are the rate constants of the pseudo-first-order and pseudo-second-order equations, respectively.

**Figure 2.**
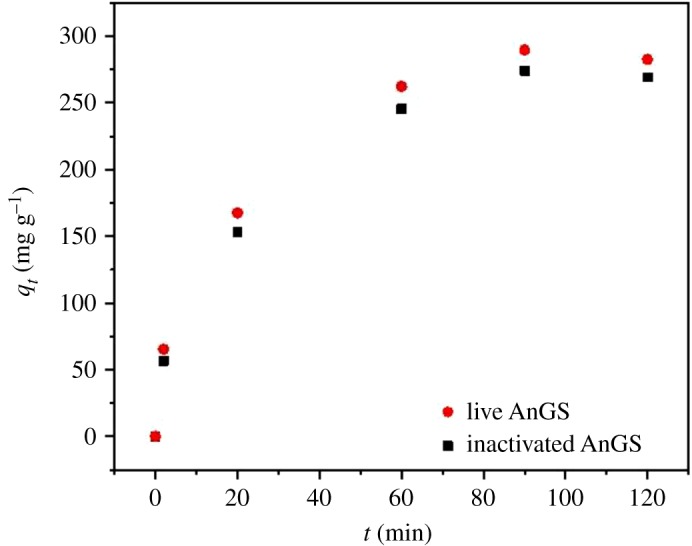
Effect of contact time for MB onto live and inactivated AnGS.

The effect of contact time on the sorption is shown in figure 2. The sorption capacities for the two absorbents increased extremely in the first 60 min because the active sites of the absorbents were vacant; and then the adsorption did not change significantly with further increase in contact time. The results also suggest no striking difference in sorption processes between live and inactivated AnGS.

As shown in table 2, the pseudo-second-order model correlates the kinetic data quite well with the higher *R*^2^ (0.99) for both live and inactivated AnGS. These results indicate that the adsorption process for both the live and inactivated AnGS could be better expressed by the pseudo-second-order model, and suggest the sorption rate is dominated by valence forces or covalent forces of chemical sorption.

**Table 2.** The adsorption kinetic constants and correlation coefficients of MB adsorption onto live and inactivated AnGS.

	pseudo-first-order			pseudo-second-order		
adsorbent	*q*_e_ (mg g^−1^)	*k*_1_ (min^−1^)	*R*^2^	*q*_e_ (mg g^−1^)	*k*_2_ (g (mg min)^−1^)	*R*^2^
live AnGS	187.90	0.011	0.87	320.51	0.0002	0.99
inactivated AnGS	176.25	0.012	0.79	312.5	0.0002	0.99

## 4. Discussion

### 4.1 Binding components and complexation mechanism

#### 4.1.1 EEM spectra

**Figure 3.**
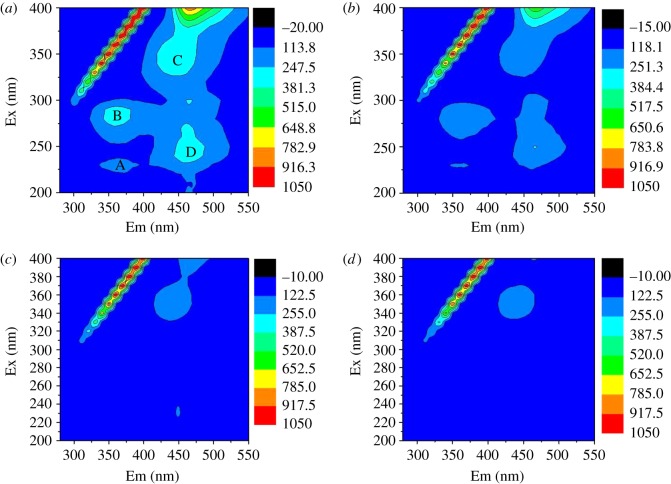
3D-EEM fluorescence spectra of EPS from live AnGS with: (*a*) 0 mg l^−1^, (*b*) 25 mg l^−1^, (*c*) 250 mg l^−1^ and (*d*) 400 mg l^−1^ MB.

To clarify the possible influence of microbial activity on biosorption, fluorescence spectra were studied using 3D-EEM and synchronous fluorescence [21]. 3D-EEM can be applied to distinguish and quantify chemical compositions of EPS extracted from live AnGS. It has been used for characterizing dissolved organic matter (DOM) in streamwater treatment [22]. The EEM spectra of the EPS samples are shown in figure 3. It was found that two main peaks of fluorescence intensity were associated with protein (PN)-like substances [15,23,24], with centre wavelengths at Ex/Em = 230/355–361 nm (peak A) and Ex/Em = 280/360–367 nm (peak B). The two peaks were similar to those reported in the literature by Sheng *et al*. [25], in a study in which EPS was extracted from aerobic and anaerobic sludge. The third and fourth main peaks were located at the Ex/Em of 350/449–454 nm (peak C) and 240/460–466 nm (peak D), which were assigned to humic acid-like and fulvic acid-like substances [26], respectively.

The fluorescence parameters of the spectra, including peak location and fluorescence intensity, are summarized in table 3. After the addition of different doses of MB, the fluorescence intensities of the four major peaks gradually decreased markedly from 156.75 to 87.67, 313.97 to 94.73, 353.27 to 155.66 and 355.32 to 92.98, respectively. This outcome suggested that the four main components of EPS were strongly quenched when MB was added, and indicated that EPS played an important role in the removal of MB during the sorption process. A similar phenomenon was reported by Shi *et al*. [27], who investigated the binding of MB to EPS in anaerobic granular sludge.

**Table 3.** Effects of MB on the peak intensity of EPS from live AnGS.

	peak A		peak B		peak C		peak D	
MB (mg l^−1^)	Ex/Em	intensity	Ex/Em	intensity	Ex/Em	intensity	Ex/Em	intensity
0	230/361.5	156.75	280/360	313.97	350/450.5	353.27	240/461.5	355.32
25	230/355.5	128.89	280/361	242.84	350/450	184.24	240/466	254.6
250	230/360	102.40	280/362	116.54	350/449.5	169.16	240/460	111.94
400	230/359	87.67	280/367	94.73	350/454	155.66	240/460	92.98

#### 4.1.2. Synchronous fluorescence

**Figure 4.**
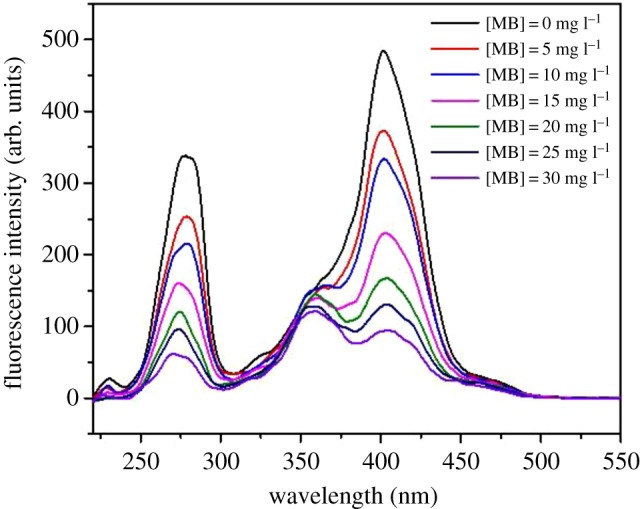
Synchronous fluorescence spectra for quenching of EPS from live AnGS titrated with MB.

This fluorescence method can be employed to investigate the interaction between ligands and proteins, and can give some information about the quenching mechanism and binding constants [28]. The technique was used to study fluorescence quenching spectra of EPS from live AnGS at various concentrations of MB, and the spectra are shown in figure 4. It was interpreted that the compound formed between MB and EPS quenched the fluorescence of EPS.

According to previous literature, the fluorescence regions corresponding to the wavelength ranges of 250–300, 300–380 and 380–550 nm could be roughly assigned to PN-like, fulvic-like and humic-like fluorescence fractions, respectively [29]. As shown in figure 4, two obvious fluorescence peaks at 280 nm (peak A) and 400 nm (peak B) were observed, and with the increase of MB concentration the fluorescence intensity decreased from 337.95 and 484.37 to 57.16 and 93.51. The result revealed that PN-like and humic-like fractions made the contribution to the binding of MB to EPS from live AnGS, which was consistent with the result of EEM.

#### 4.1.3. Quenching mechanism

Quenching mechanisms are usually divided into dynamic and static quenching [30]. Dynamic quenching is due to the collision between fluorophore and quencher whereas static quenching is attributed to the complexation between fluorophore and quencher [31,32]. Fluorescence quenching data are usually analysed according to the Stern–Volmer equation (4.1):

4.1 $$\frac{F_{0}}{F}=1+K[Q]=1+k_{q}\tau_{0}[Q],$$

where *F*_0_ and *F* are the fluorescence intensity in the absence and presence of MB, respectively, *K* is the Stern–Volmer quenching rate constant, *k*_q_ is the quenching rate constant of the biological macromolecule, *τ*_0_ refers to the lifetime of fluorescence(s), which is taken as 10^−8^ s, and [*Q*] is the MB concentration.

**Figure 5.**
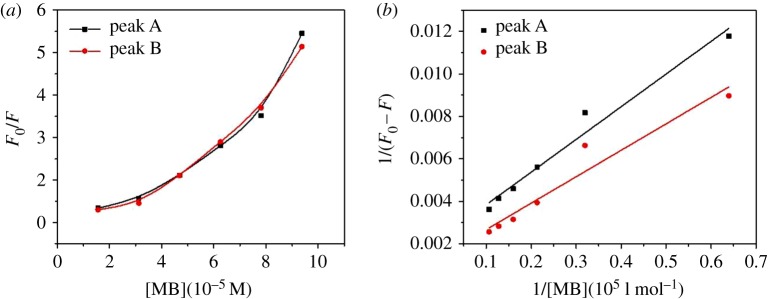
(*a*) Stern–Volmer and (*b*) modified Stern–Volmer plots of fluorescence emission for quenching of EPS from live AnGS titrated with MB.

Generally, linear plots can be concluded to indicate a single dynamic or static quenching process, whereas nonlinear plots suggest that a combined quenching (static and dynamic) process occurred [33]. The Stern–Volmer plots of the fluorescence quenching of EPS from live AnGS by MB are presented in figure 5*a*. It is apparent that the plot is an upward curve at high concentrations, which indicates that both dynamic and static quenching may be involved in the fluorescence quenching of peaks A and B. In addition, the much larger values of *k*_q_ than 2.0 × 10^10^ l mol^−1^ S^−1^ also indicate the static quenching process [34].

The static fluorescence quenching mechanism was further depicted by using the modified Stern–Volmer equation (4.2) [35]:

4.2 $$\frac{1}{\left(F_{0}-F\right)}=\frac{1}{F_{0}K_{a}[\text{MB}]}+\frac{1}{F_{0}}.$$

where *k*_a_ is the effective quenching constant. A linear relationship (*R*^2^ > 0.93) between 1/(*F_0_* − *F*) and 1/[MB] was observed for both peak A and peak B. The high binding constants (*K*_a_, 10^4^–10^5^ M^−1^) and the data from the quenching experiments confirmed that static quenching was the predominant form throughout the EPS–MB quenching (figure 5*b*).

### 4.2. FTIR

**Figure 6.**
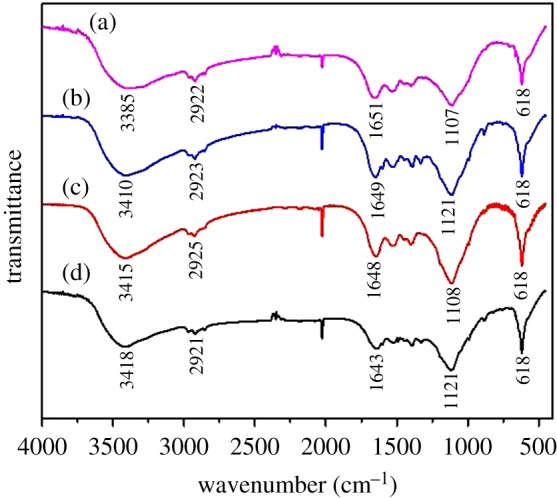
FTIR spectra of (a) live AnGS, (b) MB-loaded live AnGS, (c) inactivated AnGS, (d) MB-loaded inactivated AnGS.

The physical structure, chemical nature and functional groups (e.g. carboxyl, amino) of the biosorbents may control the biosorption performance [36]. The FTIR spectra of live and inactivated AnGS before and after MB biosorption in the range of 4000–400 cm^−1^ are demonstrated in figure 6. It was shown that the strong bands between 3418–3385 cm^−1^ implied O–H and N–H stretching vibrations of hydroxyl and amine groups [37], and the band at 2925–2921 cm^−1^ could be assigned to an asymmetric vibration of CH_2_; a distinct band at 1651–1643 cm^−1^ was associated with the stretching vibration of C–O and C–N (amide I) peptidic bond of protein, bands between 1121–1107 cm^−1^ were the result of the vibrational stretchings of O–H and C–O (carbohydrates) and the fingerprint region of the peaks at 618 cm^−1^ indicated monosubstituted aromatic structures.

As shown in figure 6, the FTIR spectra before and after MB sorption showed only slight differences. After exposure to MB, the band intensities at 1651 and 1648 cm^−1^ (amide I) for live and inactivated AnGS, respectively, slightly decreased, indicating the important role of amino groups from protein in the sorption process. The band intensities at 3385 and 1107 cm^−1^ for live AnGS, 3415 and 1108 cm^−1^ for inactivated AnGS all increased after MB biosorption, demonstrating that O–H and N–H of hydroxyl and amine, and the C–O of carbohydrates played significant parts in the sorption of MB. It was speculated that the hydroxyl and amino groups were the main binding sites in the biosorption process.

## 5. Conclusion

The sorption of MB on both the live and inactivated AnGS could be well described by the Langmuir model and followed the pseudo-second-order model. EPS binding to MB during the sorption process was explored by using 3D-EEM and synchronous fluorescence spectra, and it was concluded that PN-like substance was the main component of AnGS and the quenching was of the static quenching type. According to the FTIR spectra, hydroxyl and amino groups in AnGS were the key functional groups for sorption. Both sorption isotherm and sorption kinetic data indicate that the sorption can be considered as mainly a physical–chemical process and that metabolically mediated diffusion in the biotreatment process is negligible.

## Supplementary Material

Appearance of AnGS

## Data accessibility

All data are provided in the main text.
