# Supplementary material for: Fluorescent component and complexation mechanism of extracellular polymeric substances during dye wastewater biotreatment by anaerobic granular sludge
Source: R Soc Open Sci. 2018 Feb 28;5(2):171445. doi: 10.1098/rsos.171445 (PMC5830752; doi:10.1098/rsos.171445)
Supplement: Appearance of AnGS [file rsos171445supp1.doc]

**Supplementary data**

**Fluorescent component and complexation mechanism of extracellular polymeric substances during dye wastewater biotreatment by anaerobic granular sludge**

Na Li ^a^, Dong Wei ^a^, Qunqun Sun ^a^, Xiao Han ^a^, Bin Du ^a^ **[[1]](#footnote-2)***, Qin Wei ^b^

*^a^ School of Resources and Environment, University of Jinan, Jinan 250022, PR China*

*^b^ Key Laboratory of Chemical Sensing & Analysis in Universities of Shandong, School of Chemistry and Chemical Engineering, University of Jinan, Jinan 250022, PR China*


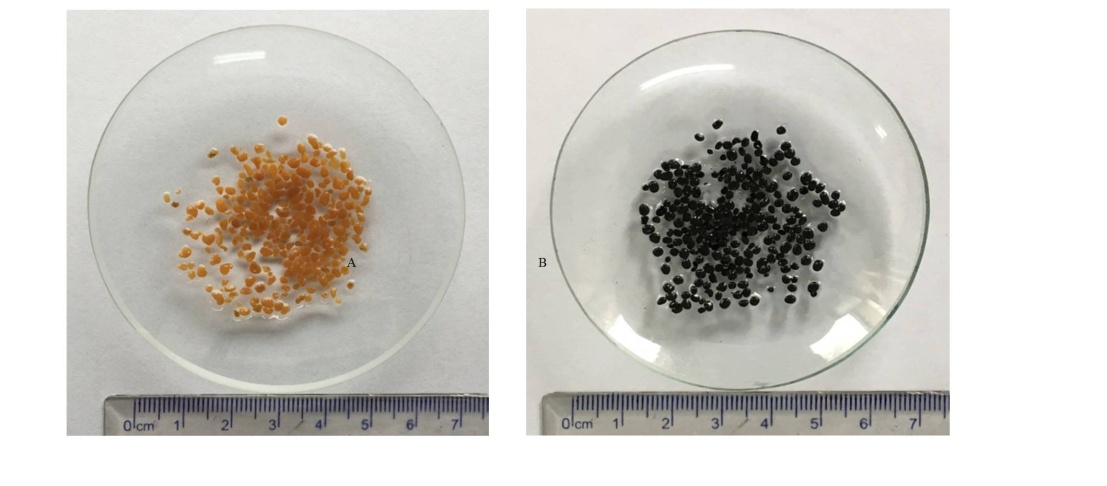


**Fig. S1** Appearance of AnGS from a WWTP.

1. ^^ Corresponding author. Tel: +86 531 8276 7370; fax: +86 531 8276 7370.

   E-mail address: dubin61@gmail.com (B. Du) [↑](#footnote-ref-2)
